# The Relationship Between Negative Life Events and Mental Health in Chinese Vulnerable Children: A Meta-Analysis Based on the Dual-Factor Model of Mental Health

**DOI:** 10.3390/bs15070882

**Published:** 2025-06-28

**Authors:** Yunrong Pu, Kuo Zhang, Xiaoliu Jiang

**Affiliations:** School of Sociology, Nankai University, Tianjin 300350, China; 2120232616@mail.nankai.edu.cn

**Keywords:** negative life events, vulnerable children, mental health, meta-analysis

## Abstract

While negative life events’(NLEs’) impact on vulnerable children’s mental health has been studied, the role of moderating variables remains unclear. Synthesizing the current evidence is critical to advancing understanding. Accordingly, this study conducted a meta-analysis utilizing a random-effects model across 77 studies, which collectively encompassed 119 effect sizes, to elucidate the connections between NLEs and mental health indicators among distressed children. The results revealed a significant positive correlation between NLEs and negative indicators of mental health (*r* = 0.42), reflecting strong and consistent associations. In contrast, NLEs were associated with a significant negative correlation with positive indicators of mental health (*r* = −0.25), reflecting weak and variable associations. Furthermore, the relationship between NLEs and the mental health of vulnerable children was found to be consistent across various age groups and subpopulations. These findings lay the groundwork for the development of targeted intervention strategies aimed at enhancing the mental well-being of vulnerable children.

## 1. Introduction

With the advancement of national governance and social assistance systems in China, significant progress has been made in safeguarding children’s rights and well-being. However, certain groups of children continue to encounter substantial challenges in their development due to complex factors such as family poverty and inadequate supervision. The term ‘vulnerable children’ (i.e., children in difficult circumstances) refers to minors who face significant challenges in daily life, healthcare, education, and social integration due to factors such as economic hardship, physical disabilities, or the absence of parental guardianship (The State Council of the People’s Republic of China, 2016). In addition to economically disadvantaged and disabled children, left-behind children (those who remain in rural settlements while one or both parents migrate to cities for employment and have not lived with them for over 6 months) and migrant children (those who have relocated with their families but lack access to stable resources) also frequently experience financial instability and insufficient parental care (The State Council of the People’s Republic of China, 2016). Consequently, some scholars consider them important subgroups of vulnerable children (N. Li & Zhuang, 2022).

As the world’s largest developing country, China has nearly 298 million children. Among them, the total number of migrant children and left-behind children reaches 138 million, accounting for 46.31 per cent of the total child population (China National Children’s Center, 2024). In addition, poor children and disabled children comprise nearly 20 per cent of the total child population (The Second National Sampling Survey on Disability Leadership Group, 2006; United Nations Children’s Fun, 2023). However, this offers abundant opportunities and samples for exploring the intersection of adversity and mental health, making such analysis more crucial and imperative. The necessity of addressing this issue is further underscored by recent government initiatives; the Ministry of Civil Affairs et al. (2023) issued Guiding Opinions on Strengthening Mental Health Care Services for Children in Difficult Circumstances in October 2023, which explicitly called for enhanced psychological support and targeted interventions for vulnerable children. In addition, within the framework of traditional Chinese culture, the group cohesion appeal of ‘family harmony and prosperity’ and the intergenerational achievement expectation of ‘expecting one’s son to be a dragon’ have led to the formation of a unique psychosocial mechanism for children in distress in China (L. Zhang, 2024; Guo et al., 2020). When they internalize this culture, the realities they face are often in structural conflict with their value expectations (Fu et al., 2025)—for example, the lack of a sense of belonging due to dysfunctional families, or the gap in intergenerational expectations due to insufficient educational resources. This discrepancy between external realities and internal expectations may further increase their susceptibility (Peng et al., 2023): externally, they are exposed to persistent risks of economic deprivation and social exclusion; internally, they are subjected to chronic psychological stress due to a crisis of value identity. This pattern of dual stressor interactions is significantly different from the developmental trajectory of the average child (F. L. Wang et al., 2019). As such, examining the mental health of vulnerable Chinese children may provide valuable insights into the psychological outcomes associated with adversity within a unique socio-cultural context.

Negative life events (NLEs) refer to adverse incidents and unfavorable changes encountered by individuals in familial, educational, or other environmental contexts, typically eliciting stress responses that necessitate adaptive coping (Chen et al., 2014). These events serve not only as significant predictors of depressive symptomatology but also constitute major risk factors for psychosomatic health disorders (Zheng et al., 2024; Karhina et al., 2023). Empirical evidence indicates a dose–response relationship: increased quantity and diversity of NLEs during childhood are associated with elevated risks of adult-onset psychiatric disorders and suicidality (Bagge et al., 2013; Cuijpers et al., 2011). Compared to typically developing peers, vulnerable children residing in high-risk environments characterized by compound adversities exhibit amplified sensitivity to the mental health consequences of NLEs, potentially due to cumulative exposure to stressors (Tong, 2020).

Despite the extant literature examining associations between NLEs and mental health outcomes among vulnerable children, three critical limitations persist in the current scholarship: First, predominant classification approaches compartmentalize these populations into discrete subgroups (e.g., left-behind versus migrant children) (L. Han et al., 2021; Q. Wang et al., 2020), thereby overlooking their shared multidimensional adversities. This fragmentation impedes the comprehensive integration of cross-group commonalities essential for delineating universal mental health patterns in this heterogenous yet interconnected population. Second, even within subgroups, there was considerable variation between studies. For example, one study showed a significant negative correlation between NLEs and psychological resilience in left-behind children (*r* = −0.36) (L. Han et al., 2019), but others showed a weaker positive correlation (*r* = 0.17) (Zhou et al., 2013). Similar divergences exist in other types of vulnerable children (K. Li et al. 2023; Yang et al., 2016). Third, conventional research paradigms predominantly link NLEs to unidimensional mental health indicators—either negative (e.g., depression) or positive (e.g., well-being)—in isolation. This approach, however, overlooks the complexity of psychological well-being. The World Health Organization (WHO) defines health as “a state of complete physical, mental, and social well-being and not merely the absence of disease or infirmity” (World Health Organization, 1948), broadening the concept beyond the absence of pathology to include positive functioning across emotional, cognitive, and social domains. Building on this holistic perspective, the Dual-Factor Model of Mental Health (DFM) posits that psychological well-being is a bidimensional construct, encompassing both the absence of psychopathology and the presence of flourishing (Keyes & Lopez, 2002).To operationalize this framework, negative indicators of mental health focus on psychopathological symptoms, such as depression, anxiety, and behavioral problems, to assess the absence of clinically significant symptoms (Achenbach et al., 2016; Suldo & Shaffer, 2008). In contrast, positive indicators of mental health measure psychological strengths and resources, including well-being, life satisfaction, psychological resilience, and social functioning, to evaluate flourishing and optimal functioning (Keyes & Ryff, 1999; Keyes, 2002). This theoretical advancement, rooted in the WHO’s definition and the DFM, necessitates multiaxial assessment frameworks that concurrently examine both positive and negative mental health domains, aligning with existing meta-analyses that underscore the importance of reducing distress while enhancing well-being for a comprehensive understanding of mental health.

These discrepancies likely stem from methodological variability (e.g., measurement tools, sample characteristics) and underscore the imperative of systematic quantitative synthesis through meta-analysis. Guided by the dual-factor framework, this meta-analytic investigation examines the magnitude and consistency of associations between NLEs and bidimensional mental health outcomes (positive and negative) in Chinese vulnerable children, while identifying critical moderators. This study aims to inform targeted policy interventions to enhance psychological resilience and social adaptation within this at-risk population.

The Transactional Model of Stress and Coping (TMSC) posits that psychological stress not only arises due to environmental demands but manifests through dynamic interactions between external stimuli and individuals’ cognitive appraisals. Central to this framework is the premise that evaluations of stressors—including perceived threat or controllability—critically shape the valence and intensity of stress responses (Lazarus & Folkman, 1984). Vulnerable children, subjected to cumulative adversities such as familial economic strain, parental absence, and social support deprivation, face heightened vulnerability to psychosocial risks (Y. Han et al., 2020; Salisbury et al., 2020). Exposure to chronic social exclusion and perceived discrimination reinforces maladaptive cognitive schemas (e.g., negative self-attributions), which, when activated by NLEs, may amplify detrimental mental health outcomes through bidirectional person–environment interactions (Abela & Skitch, 2007). Empirical findings corroborate this pathway: the increased frequency of NLEs in this population correlates strongly with elevated risks of internalizing (e.g., anxiety, depression) and externalizing (e.g., aggression, conduct issues) psychological disorders (Q. Wang et al., 2020; F. Li & Jhang, 2023). Therefore, this study proposes H1: NLEs exhibit significant positive associations with adverse mental health indicators among vulnerable children.

The Conservation of Resources (COR) theory posits that psychological distress arises when resource demands exceed available reserves, with resource depletion exerting stronger adverse effects than resource accumulation affords benefits (Hobfoll, 1989). Vulnerable children—characterized by disproportionately scarce social capital (e.g., stable caregiving networks) and psychological reserves (e.g., coping strategies)—confront amplified resource–environment imbalances compared to normative populations (Tong, 2020; Shi & Fu, 2021). NLEs operate as chronic resource drains within this framework, triggering three interrelated pathways: (1) Resource Depletion, which disrupts homeostasis between environmental challenges and adaptive capacities (Chen et al., 2014); (2) Affective Dysregulation, which exacerbates psychological conflicts and negative affectivity through prolonged stress activation (Zheng et al., 2024); (3) Eudaimonic Erosion, which diminishes positive affect and erodes protective resources (e.g., optimism), thereby progressively reducing life satisfaction and subjective well-being (X. Li et al., 2023). Therefore, this study also proposes H2: NLEs demonstrate robust negative associations with positive indicators of mental health in vulnerable children.

TMSC and COR can complement each other in elucidating the different but related mechanisms of action by which negative life events affect the mental health of vulnerable children. TMSC emphasizes the role of cognitive patterns formed through an individual’s long-term adaptation to their environment (Lazarus & Folkman, 1984), making it particularly suitable for analyzing how negative cognitive tendencies among vulnerable children may lead to adverse psychological outcomes when coping with adversity. In contrast, COR theory focuses on the acquisition and depletion of psychological resources (Hobfoll, 1989), highlighting how the persistent lack of both material and psychological resources can limit the adaptive capacity of vulnerable children, resulting in cumulative psychological distress. This theoretical perspective is especially relevant for understanding the negative mental health consequences stemming from resource deficits. Compared to more macro-level theories, such as the ecological systems theory or cumulative risk models (Evans et al., 2013; Bronfenbrenner, 1979), TMSC and COR not only integrate intrapersonal cognitive mechanisms and the influence of environmental resources, but also allow for a multi-level analysis of why vulnerable children are more vulnerable to psychological harm from negative life events. Therefore, combining these two frameworks provides a more nuanced and mechanistically explicit theoretical foundation for comprehensively understanding the broad and profound impacts of negative life events on various dimensions of mental health among vulnerable children.

### Moderator Variable

Type of Mental Health Indicator: In studies that used negative indicators of mental health as outcome variables, the correlations between NLEs and indicators such as depression and psychological symptoms were relatively high. For example, Xiong’s study found a strong correlation (*r* = 0.60) between NLEs and depression in poor children (H. Xiong et al., 2016); however, the correlation between NLEs and indicators such as behavioral problems and internalized emotional problems was relatively low. For example, Li’s study found a weak correlation between NLEs and left-behind children’s behavioral problems (*r* = 0.17) (F. Li & Jhang, 2023). Similarly, NLEs had relatively high correlations with positive indicators such as social support, psychological resilience, and subjective well-being (L. Han et al., 2021; X. Li et al., 2023), and relatively low correlations with indicators such as self-esteem, psychological capital, and adaptive behavior (Keyes & Lopez, 2002; Gao et al., 2019). Therefore, Hypothesis 3 is proposed: The different types of outcome variable significantly moderate the NLEs–mental health association in vulnerable children.

Type of NLEs: The psychological impact of specific adverse life event sources on vulnerable children’s mental health remains inconsistent across empirical studies. For instance, divergent findings emerge in research on left-behind children: while some scholars identify interpersonal conflicts as the most influential factor (with correlation coefficients in the range (*r* = 0.29~0.55) (Zhou et al., 2013; H. Wang & Liu, 2018), others highlight academic pressure as demonstrating stronger associations (*r* = 0.25~0.64) (Huang et al., 2016; Liao et al., 2015). This empirical discrepancy reveals there is insufficient understanding regarding the differential effects of adverse event typologies. Therefore, this study proposes H4: The source of NLEs plays a moderating role between NLEs and mental health in vulnerable children.

Educational stage: Vulnerable children of different ages may encounter different types of NLEs, and due to variations in their psychological development and coping strategies, the extent to which their mental health is affected may also differ. For example, younger children, such as those in elementary school, have been found to encounter disciplinary events more frequently, while adolescents in middle school are predominantly subjected to academic stress (Shi & Fu, 2021). Furthermore, developmental differences may alter how children perceive and respond to adverse experiences, with younger children possibly lacking effective coping strategies compared to older students. These findings indicate that the impact of NLEs on psychological well-being may manifest differently across educational stages. Therefore, this study proposes H5: Educational stage moderates the relationship between NLEs and mental health outcomes in vulnerable children.

Categories of Vulnerable Children: Different subgroups of vulnerable children may experience and respond to negative life events (NLEs) in distinct ways. For example, economically disadvantaged children are more likely to exhibit social withdrawal due to unfavorable family socioeconomic conditions, which can exacerbate their psychological distress (C. Wang & Yang, 2016). In contrast, disabled children may experience delays in neurocognitive development, making them more susceptible to the negative consequences of disciplinary incidents (Shi & Fu, 2021). These subgroup differences underscore the importance of distinguishing among categories of vulnerable children when exploring the impact of NLEs. Accordingly, this study proposes H6: The category of vulnerable children moderates the relationship between NLEs and mental health outcomes.

## 2. Materials and Methods

### 2.1. Literature Search

A comprehensive search was conducted of both the English and Chinese literature. All English search terms were translated into standardized Chinese psychological terminology by professional translators. These validated Chinese equivalents were then used as search terms in the Chinese databases, applying the same search logic as in the English-language search. The Chinese literature was searched through the China National Knowledge Infrastructure (CNKI), China Maste’s Theses Full-text Database (CMFD), China Doctoral Dissertations Full-text Database (CDFD) and Wanfang Database. The phrase “negative life events” was also matched with “migrant children”, “left-behind children”, “children in poverty”, and “children with disabilities”, and then combined with “mental health”, “depression”, “anxiety”, “behavioral problems”, “social support”, and “life satisfaction”. Additionally, terms such as “adaptive behavior”, “mental resilience”, “mental capital”, “happiness”, “self-esteem”, “internalisation problems” and “sense of security” were used to search for research containing these keywords. Then, we manually checked the reference lists of selected studies and reviewed articles to reduce the likelihood of missing relevant studies.

**Figure 1:**
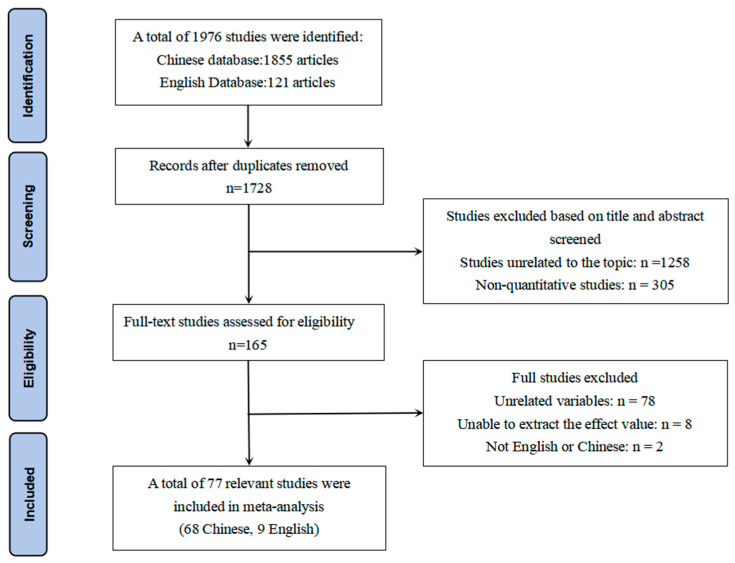
PRISMA literature screening diagram.

The English literature was searched through Springer, EBSCO, PubMed, Web of Science, and Google Scholar. The terms “negative life events”, “migrant children”, and “left-behind children” were used. The terms “children living in poverty”, “children with disabilities” and “disabled children” were also included, along with “mental health”, “depression”, “anxiety”, “behavioral problems”, and “social problems”. Furthermore, “behavioral problems”, “social support”, “life satisfaction”, “adaptive behavior”, “resilience”, “psychological capital”, “happiness”, and “self-esteem” were used to search for research containing these keywords. A snowball search was conducted to avoid omissions, and the literature was searched from January 2000 to January 2024, taking into account that empirical research on children in distress was mainly published after 2000. The literature was screened according to the PRISMA guidelines, as shown in Figure 1.

### 2.2. Literature Inclusion and Exclusion Criteria

(1) The literature comprises empirical research and reports the correlation coefficient r or a statistic that can be transformed (e.g., t-tests, F-tests, chi-square); (2) the study population m vulnerable children in China; (3) the research instrument is clearly stated and basic information about the questionnaire used in the study (including reporting Cronbach’s α coefficients and full scale names) is clearly listed in the methodology section; (4) only one of the data is taken as duplicated in the publication; (5) if the dissertation has been published publicly in journals and the research data is reported, the journal paper is selected for analysis; (6) if more than one independent samples are reported in the same piece of literature, they were coded separately.

### 2.3. Literature Coding

Each study was coded according to the following information: literature information, correlation coefficient, sample size, type of subjects, school age group, and type of outcome variable (N. Li & Zhuang, 2022; L. Han et al., 2019). Each independent sample was coded once, taking the data as a whole when the variable was reported at the overall level, or taking the mean at the dimensional level if it was reported at the sub-dimensional level (Lipsey & Wilson, 2001). Coding was carried out independently by two coders and the coding consistency was 93.6%; inconsistent codes were checked and agreed upon.

### 2.4. Statistical Analyses

(1) CMA 3.0 was used to perform a meta-analysis of the data to determine whether there was publication bias, using a combination of fail-safe N and Egger’s test, and heterogeneity was assessed using *Q* values and *I*^2^.The Q-test is a test of significance for the sum of the weighted squared differences between each effect size and the overall estimated effect size, and if significant (*p* < 0.05), then significant heterogeneity between the effect sizes is considered to exist and a random-effects model should be used (Cheung, 2014; Higgins et al., 2003). The larger the *I*^2^ statistic, the more significant the heterogeneity, with *I*^2^ values of 25%, 50%, and 75% indicating low, medium and high levels of heterogeneity, respectively, and high levels of heterogeneity being more appropriate for choosing a random effects model (Higgins et al., 2003). (2) A random effects model was used for the main effects test, and sensitivity analyses were performed using the ‘remove-one’ method. (3) A relative weight analysis was conducted using RWA web to investigate the differences in the predictive effects of different sources of NLEs on mental health. (4) The moderating effect was tested using subgroup analysis.

## 3. Results

### 3.1. Literature Inclusion and Quality Assessment

Data validity, measurement tool reliability, and other indicators were selected as evaluation criteria, with a total score of 10 points: the higher the score, the better the quality of the article (H. Xiong et al., 2016). The results showed that the mean value of the scores for literature quality assessments was 6.35, and only seven documents scored lower than the theoretical mean value of 5, indicating that the overall quality of the literature was relatively good.

### 3.2. Publication Bias Test

As shown in Table 1, the insecurity coefficients of NLEs and positive and negative mental health are 65,319 and 89,069, respectively, indicating that the current results are robust. The results of Egger’s test for positive indicators of mental health and negative indicators of mental health were not significant. In conclusion, there is no serious publication bias in this study.

### 3.3. Heterogeneity Test

As shown in Table 2, the Q-value between the variables in each group reached the significant level and the *I*^2^ values were high, so the random effects model was used in all subsequent analyses. The *I*^2^ values were 95.18% and 96.25%, which implies that 95.18% and 96.25% of the variance originated from the real differences between the effect values, i.e., the between-study variance not only came from sampling errors but was also affected by the between-group error. This suggests the need to further explore the moderating variables affecting the relationship between the two.

### 3.4. Main Effects Test

As shown in Table 3, NLEs were significantly negatively correlated with positive indicators of mental health (*r* = −0. 25, *p* < 0.001) and significantly positively correlated with negative indicators of mental health (*r* = 0. 42, *p* < 0. 001). Referring to Lipsey’s criterion (Lipsey & Wilson, 2001), *r*-values of 0.10, 0.25, and 0.40 correspond to low, medium, and high effect sizes, respectively, which suggests a weak negative correlation between NLEs and indicators of positive mental health and a moderate positive correlation with indicators of negative mental health. Sensitivity analyses showed that after excluding any one sample, the *r*-values fluctuated between −0.252 and −0.242, 0.422 and 0.436, respectively, which indicated that the estimation results had a high degree of stability.

### 3.5. Relative Weight Analysis

After completing the main effects test on overall NLEs–mental health associations, we performed a secondary extraction aimed at identifying studies that explicitly reported Pearson correlations among the individual dimensions of negative life events. For each eligible study, we recorded the published r values exactly as reported; no coefficients were estimated or transformed from other statistics. These inter-dimension coefficients—covering interpersonal issues, academic stress, punishment, loss, health adaptation, and other stressors—were then assembled into a pooled correlation matrix, as shown in Table 4. Subsequently, we conducted a relative weight analysis of this matrix. As shown in Table 5, relationship problems were the strongest predictor of negative mental health, explaining 32.20% of the variance. Among the predictors of positive mental health, other stresses (including ”hating school”, ”relationship problems”, “fights”, and “scolded by parents” events) were the strongest predictors of positive mental health, explaining 36.80 per cent of the variance.

### 3.6. Analysis of Moderating Effects

As shown in Table 6, we examined the moderating effects for each moderator variable. The types of vulnerable children included left-behind, migrant, impoverished, and disabled children; the mental health outcomes were classified as composite symptoms, behavioral issues, depression, anxiety, and other indicators; educational stage was divided into primary school, secondary school, and combined schools; in addition, the source of negative life events was categorized into interpersonal issues, academic stress, punishment, loss, health adaptation, and other stressors. The moderating effects of type of vulnerable children, type of mental health indicator, and educational stage on the relationship between NLEs and negative indicators of mental health were not significant (*Q_b_* = 3.08, 4.97, 0.07, *p* > 0.05). The moderating effect of sources of NLEs was significant (*Q_b_* = 26.89, *p* < 0.001), which suggests that there are differences in the strength of the association between different sources of NLEs and negative mental health. Specifically, the highest correlation was found between interpersonal issues and negative mental health (*r* = 0.46), followed by academic stress (*r* = 0.42). The correlations between negative mental health and other stressors, punishment, health adaptation, and loss were 0.38, 0.34, 0.34, and 0.28, respectively.

As shown in Table 7, type of mental health indicator and the source of NLEs had a significant moderating effect on the relationship between NLEs and positive mental health (*Q_b_* = 21.55, *p* = 0.003; *Q_b_* = 18.85, *p* = 0.002), suggesting that (1) the strength of the association between NLEs and positive mental health varied with the type of outcome variable, and the association between NLEs and the psychological capital, an outcome variable, had the strongest correlation (*r* = −0.39); and (2) the strength of the association between different NLEs and positive mental health varied, with other stresses having the strongest correlation with positive mental health (*r* = −0.25).

## 4. Discussion

This meta-analysis examined the relationship between NLEs and mental health outcomes among Chinese vulnerable children within the dual-factor model of mental health. The findings revealed a moderate correlation (*r* = 0.42) between NLEs and negative indicators of mental health, alongside a weaker yet significant correlation (*r* = −0.25) with positive psychological constructs. Relative weight analyses identified interpersonal conflicts and academic stress as the most salient stressors impacting mental health, while moderation analyses highlighted source-specific effects of adversity (e.g., family-related vs. school-related events) and differential impacts across outcome types (e.g., psychological distress vs. adaptive functioning). These results address critical gaps in the existing literature by systematically differentiating outcome heterogeneity and integrating multidimensional mental health frameworks. The findings underscore the importance of targeted interventions to mitigate cumulative adversity and strengthen psychological resources in this population.

### 4.1. Main Effects of Negative Life Events on Mental Health in Vulnerable Children

The main effects analysis showed that NLEs experienced by vulnerable children were moderately positively correlated with negative indicators of mental health and weakly negatively correlated with positive indicators of mental health, verifying Hypotheses 1 and 2. The above results were in line with the expectations derived from the stress interaction model and resource preservation theory (Lazarus & Folkman, 1984; Hobfoll, 1989). At the practical level, vulnerable children face multiple real-life challenges, such as family economic pressure and lack of social support; at the psychological level, their positive coping and socio-emotional abilities are relatively low, and they are more likely to form maladaptive negative schemas (Abela & Skitch, 2007). Therefore, these risk susceptibilities interact with NLEs when they are encountered, further reducing the psychological resources needed by vulnerable children, which in turn adversely affects mental health. Previous meta-analyses have identified perceptions of discrimination as an important risk factor for the mental health of children in difficult circumstances (N. Li & Zhuang, 2022); the current study extends the findings of previous research and suggests that special attention should be paid to children in difficult circumstances who have a higher accumulation of NLEs and higher levels of perceptions of discrimination when assessing and monitoring their mental health.

The results of the relative weighting analyses show that interpersonal relationship problems, academic pressure, and other stresses that include intimate relationship conflicts have a greater impact on both negative and positive indicators of mental health for children in difficulty. Therefore, psychological support for children in difficult circumstances should, on the one hand, pay more attention to their interpersonal problems, academic pressure, family conflicts, and other major stressors, and, on the other hand, cultivate socio-emotional competence and positive coping styles, reduce perceptions of discrimination, and improve their academic and interpersonal adaptation levels. Previous studies on the life events of ordinary children have also found that interpersonal problems and academic stress are the main stressors affecting children’s mental health (Miao, 2020). This suggests that although children in difficult circumstances experience higher levels of life event stress than children in the general population (Shi & Fu, 2021), the main developmental tasks and stressors faced by both are consistent. In addition, the study found that ‘loss’ had a relatively low impact on the psychological well-being of children in difficult circumstances. This suggests that although ‘loss’ is considered to be one of the most serious NLEs, it is not a major stressor for children in difficult circumstances due to the relatively low frequency of such events and their overall limited impact on children’s psychological well-being.

### 4.2. Moderating Role of Correlates in the Relationship Between Negative Life Events and Mental Health Among Vulnerable Children

The study found that NLEs exhibited strong and consistent correlations with all indicators of negative mental health in vulnerable children. Specifically, NLEs demonstrated moderate to high effect sizes on anxiety, depression, behavioral problems, and overall psychological symptoms. However, the correlations between NLEs and positive indicators of mental health showed heterogeneity: NLEs exerted stronger influences on psychological capital and well-being, but weaker effects on social support, self-esteem, and adaptive behaviors. These results partially validated Hypothesis 3. This indicates that NLEs serve as critical risk factors for emotional and behavioral problems in vulnerable children (Q. Wang et al., 2020; F. Li & Jhang, 2023), with their impacts on negative mental health demonstrating transdiagnostic commonalities. Therefore, psychological interventions for these children should prioritize comprehensive planning to establish integrated social and psychological support systems. Additionally, the differential correlations between NLEs and positive indicators of mental health suggest that intervention strategies should not only focus on building social support networks to leverage their buffering effects but also adopt a strengths-based approach to cultivate psychological capital and resilience. Enhancing positive psychological resources through their regulatory and cumulative effects may further promote psychological and social adaptation in this population (Xiao et al., 2024; J. Xiong et al., 2020).

Moderation analyses revealed systematic variations in the impacts of different types of NLEs. Interpersonal conflicts, academic stress, and other trauma-related events showed stronger associations with both negative and positive mental health outcomes, whereas losses exhibited weaker correlations. These findings support Hypothesis 4 and align with results from relative weight analyses. Previous meta-analyses on life events in general child/adolescent populations similarly identified academic stress and interpersonal conflicts as having the strongest associations with depression, contrasted with minimal effects from losses (Miao, 2020).

School-age stages did not significantly moderate the relationship between NLEs and mental health, indicating that such events exert consistent impacts across developmental periods. Similarly, no significant moderating effects were observed for subtypes of vulnerable children (i.e., left-behind, migrant, impoverished, or disabled children). Correlations between NLEs and negative mental health ranged from 0.40 to 0.48 across subtypes, while correlations with positive mental health ranged from −0.16 to −0.26. Although the group differences did not reach statistical significance, the results indicated that stronger associations emerged between NLEs and negative mental health in disabled and impoverished children compared to left-behind and migrant children. This pattern implies that disabled and impoverished children may face heightened life stressors and psychological vulnerability, necessitating targeted interventions that address not only basic subsistence needs through welfare programs but also enhanced psychosocial support.

### 4.3. Implications

The findings of this study suggest that prioritizing interventions in the areas of interpersonal relationships and academic stress may help improve the mental health of vulnerable children in China. Schools could consider structured initiatives such as conflict resolution workshops or class discussions to address interpersonal issues, and provide academic counseling or stress management support to those facing academic pressures (Yuan et al., 2024). We also found a notable link between negative life events and lower psychological capital and well-being, indicating that interventions to build resilience, optimism, and coping skills—such as evidence-based social-emotional learning (SEL) programs—may be beneficial, especially for children experiencing frequent stress (Jones & Doolittle, 2017). Given that these patterns are consistent across different subgroups and age groups, making mental health services broadly accessible to all vulnerable children, rather than targeting only certain groups, may be advantageous (Yuan et al., 2024). Establishing multi-tiered support systems and offering relevant teacher training can help enable timely assistance to be provided for those in need.

### 4.4. Study Limitations and Future Directions

(1) Due to the limited number of published studies, our analysis of associations between NLEs and positive indicators of mental health did not include samples of disabled children, meaning that data on positive mental health among disabled children were missing. Future studies should broaden their scope to comprehensively evaluate the impact of NLEs on both negative and positive mental health across all subgroups of vulnerable children, with particular attention to including disabled children in the analysis. (2) This study did not thoroughly explore the mediating mechanisms through which NLEs influence mental health in vulnerable children, constraining a deeper understanding of their psychological adaptation processes. Subsequent research should identify and test potential mediating variables to systematically elucidate the intrinsic mechanisms underlying these relationships. (3) As the current meta-analysis is based solely on cross-sectional studies, our findings provide only static correlational evidence, which limits the ability to draw conclusions about causality. Therefore, we encourage future research to conduct meta-analyses of longitudinal studies, which would help to clarify the dynamic processes and provide stronger evidence for causal relationships between negative life events and mental health outcomes among vulnerable children.

## 5. Conclusions

The current study revealed that: (1) NLEs demonstrated moderate positive correlations with negative indicators of mental health and weak negative correlations with positive indicators of mental health in Chinese vulnerable children. (2) NLEs related to interpersonal conflicts and academic stress emerged as critical risk factors for mental health outcomes in this population. (3) Different negative indicators of mental health did not significantly moderate the association between NLEs and negative mental health. In contrast, the type of positive indicators had a significant effect on the relationship between NLEs and positive mental health, with psychological capital and subjective well-being showing stronger links than social support, self-esteem, and adaptive behaviors. (4) The relationships between NLEs and mental health did not significantly differ across school-age stages or subtypes of vulnerable children. These findings underscore the importance of implementing targeted, evidence-based interventions that address key sources of psychological distress, particularly interpersonal conflicts and academic pressure, among vulnerable children. Future studies should examine the long-term effectiveness of such interventions and explore the mechanisms through which positive psychological resources, such as psychological capital and subjective well-being, can be enhanced to buffer the impact of negative life events. Policymakers and practitioners are encouraged to develop and sustain accessible mental health support systems tailored to the diverse needs of vulnerable children in China.

## Figures and Tables

**Table 1:** Results of publication bias test.

Variable	*k*	*n*	Fail-Safe	Intercept	95% Confidence Interval		Two-Tailed Test	
					LL	UL	*t*	*p*
Negative Mental Health	51	40,214	89,069	0.38	−3.32	4.09	0.21	0.843
Positive Mental Health	69	74,594	65,319	1.51	−0.54	3.56	1.47	0.151

Note: *k* is the number of studies included in the analysis; *n* is the total sample size of *k* samples; fail-safe is the fail-safe coefficient.

**Table 2:** Heterogeneity test results.

Variable	*k*	Heterogeneity *Q* Test				Tau-Squared			
		*Q*	*df (Q)*	*p*	*I* ^2^	Tau-Squared	SE	Variance	Tau
Negative Mental Health	51	1308.23	49	<0.001	96.25%	0.03	0.01	0	0.18
Positive Mental Health	69	1411.75	68	<0.001	95.18%	0.02	0.01	0	0.14

Note: *k* is the number of studies; *Q*-value and *p*-value are the test statistic and the risk to be taken in rejecting the original hypothesis in the *Q*-test, respectively.

**Table 3:** Random effects model test results.

Variable	*k*	*r*	95% Confidence Interval		Two-Tailed Test	
			LL	UL	*Z*	*p*
Negative Mental Health	50	0.42	0.38	0.46	16.98	<0.001
Positive Mental Health	69	−0.25	−0.28	−0.21	−14.27	<0.001

Note: *k* = number of studies included in the analysis.

**Table 4:** Correlation matrix of variables in relative weight analysis.

Variable	1	2	3	4	5	6
1. Interpersonal problems (*K*, *N*)	1	0.63 *** (5, 3286)	0.65 *** (5, 3286)	0.41 *** (5, 3286)	0.52 *** (5, 3286)	0.55 *** (5, 3286)
2. Study stress (*K*, *N*)	0.63 *** (8, 4019)	1	0.65 *** (5, 3286)	0.37 *** (5, 3286)	0.50 *** (5, 3286)	0.48 *** (5, 3286)
3. Punishment (*K*, *N*)	0.63 *** (8, 4019)	0.62 *** (8, 4019)	1	0.47 *** (5, 3286)	0.53 *** (5, 3286)	0.70 *** (5, 3286)
4. Loss (*K*, *N*)	0.50 *** (8, 4019)	0.46 *** (8, 4019)	0.56 *** (8, 4019)	1	0.47 *** (5, 3286)	0.34 *** (5, 3286)
5. Health adaptation (*K*, *N*)	0.52 *** (8, 4019)	0.53 *** (8, 4019)	0.54 *** (8, 4019)	0.53 *** (8, 4019)	1	0.50*** (5, 3286)
6. Other stresses (*K*, *N*)	0.57 *** (6, 3068)	0.50 *** (6, 3068)	0.71 *** (6, 3068)	0.46 *** (6, 3068)	0.55 *** (6, 3068)	1

Note: *K* is the number of effect sizes, *N* is the sample size, and *** stands for *p* < 0.001. The correlation matrix between negative mental health and negative life events is shown in the lower left corner, and the correlation matrix between positive mental health and negative life events is shown in the upper right corner.

**Table 5:** Relative weighting analysis of negative/positive mental health.

Independent Variable	Negative Mental Health		Positive Mental Health	
	*W*	*%R* ^2^	*W*	*%R* ^2^
Interpersonal Problems	0.08	32.20	0.02	23.84
Academic Stress	0.06	24.21	0.01	14.91
Punishment	0.02	8.62	0.01	11.17
Loss	0.02	6.33	0.00	2.44
Health Adaptation	0.02	11.37	0.01	10.84
Other Stressors	0.04	17.27	0.03	36.80
*R* ^2^	0.26		0.07	

Note: *W* denotes the relative weight of each variable; *R*^2^ denotes the variance explained by the six variables together; and *%R*^2^ indicates the contribution of each variable to the total explained variance.

**Table 6:** Results of the moderating effect test.

Moderator	Heterogeneity Test			Category	*k*	95% CI			Two-Tailed Test	
	*Q_b_*	*df*	*p*			Point Estimate	LL	UL	*Z*	*p*
Type of Vulnerable Children	3.08	3	0.381	Left-Behind	31	0.40	0.35	0.46	12.84	<0.001
				Migrant	9	0.41	0.30	0.51	6.88	<0.001
				Impoverished	8	0.50	0.40	0.59	8.22	<0.001
				Disabled	3	0.48	0.25	0.66	3.80	<0.001
Type of Outcome Variable	4.97	4	0.294	Composite Symptoms	18	0.45	0.38	0.52	11.04	<0.001
				Behavioral Issues	7	0.31	0.18	0.43	4.46	<0.001
				Depression	17	0.45	0.37	0.52	10.18	<0.001
				Anxiety	5	0.42	0.27	0.54	5.35	<0.001
				Other	4	0.38	0.20	0.53	4.05	<0.001
Educational Stage	0.07	2	0.972	Primary School	3	0.42	0.22	0.58	3.98	<0.001
				Secondary School	29	0.46	0.36	0.55	8.05	<0.001
				Combined Schools	18	0.41	0.34	0.49	9.72	<0.001
Source of Negative Life Events	26.89	5	<0.001	Interpersonal Issues	26	0.46	0.41	0.50	15.83	<0.001
				Academic Stress	26	0.42	0.37	0.47	14.30	<0.001
				Punishment	26	0.34	0.29	0.39	11.45	<0.001
				Loss	25	0.28	0.22	0.34	9.03	<0.001
				Health Adaptation	26	0.34	0.29	0.40	11.48	<0.001
				Other Stressors	22	0.38	0.32	0.43	11.76	<0.001

Note: *k* is the number of studies included in the analysis.

**Table 7:** Results of the moderating effect test.

Moderator	Heterogeneity Test			Category	*k*	95% CI			Two-Tailed Test	
	*Q_b_*	*df*	*p*			Point Estimate	LL	UL	*Z*	*p*
Type of Vulnerable Children	3.77	3	0.293	Left-Behind	50	−0.26	−0.30	−0.22	−12.77	<0.001
				Migrant	13	−0.25	−0.32	−0.17	−6.21	<0.001
				Impoverished	5	−0.16	−0.28	−0.03	−2.35	<0.001
Type of Outcome Variable	21.55	7	0.003	Social Support	15	−0.18	−0.25	−0.12	−5.35	<0.001
				Life Satisfaction	4	−0.32	−0.43	−0.20	−4.90	<0.001
				Adaptive Behavior	6	−0.21	−0.31	−0.20	−8.44	<0.001
				Psychological Resilience	19	−0.26	−0.32	−0.20	−8.44	<0.001
				Psychological Capital	5	−0.39	−0.48	−0.28	−6.65	<0.001
				Well-Being	7	−0.36	−0.44	−0.26	−7.16	<0.001
				Self-Esteem	7	−0.22	−0.31	−0.12	−4.34	<0.001
				Other	6	−0.15	−0.25	−0.04	−2.62	<0.001
Educational Stage	1.32	2	0.521	Primary School	4	−0.32	−0.44	−0.18	−4.42	<0.001
				Secondary School	46	−0.25	−0.29	−0.21	−11.60	<0.001
				Combined Schools	19	−0.23	−0.29	−0.17	−7.14	<0.001
Source of Negative Life Events	18.85	5	0.002	Interpersonal Problems	25	−0.23	−0.28	−0.18	−8.74	<0.001
				Academic Stress	25	−0.21	−0.26	−0.16	−7.69	<0.001
				Punishment	25	−0.19	−0.24	−0.14	−7.12	<0.001
				Loss	25	−0.10	−0.16	−0.05	−3.81	<0.001
				Health Adaptation	25	−0.17	−0.23	−0.12	−6.44	<0.001
				Other Stressors	23	−0.25	−0.30	−0.20	−8.92	<0.001

Note: *k* is the number of studies included in the analysis.

## Data Availability

Data are available from the corresponding author upon reasonable request.
